# Supplementary material for: Quantification of Iron Release from Native Ferritin and Magnetoferritin Induced by Vitamins B2 and C
Source: Int J Mol Sci. 2020 Aug 31;21(17):6332. doi: 10.3390/ijms21176332 (PMC7504058; doi:10.3390/ijms21176332)
Supplement: Supplementary file 1 [file ijms-21-06332-s001.pdf]

International Journal of *Molecular Sciences*

MDPI

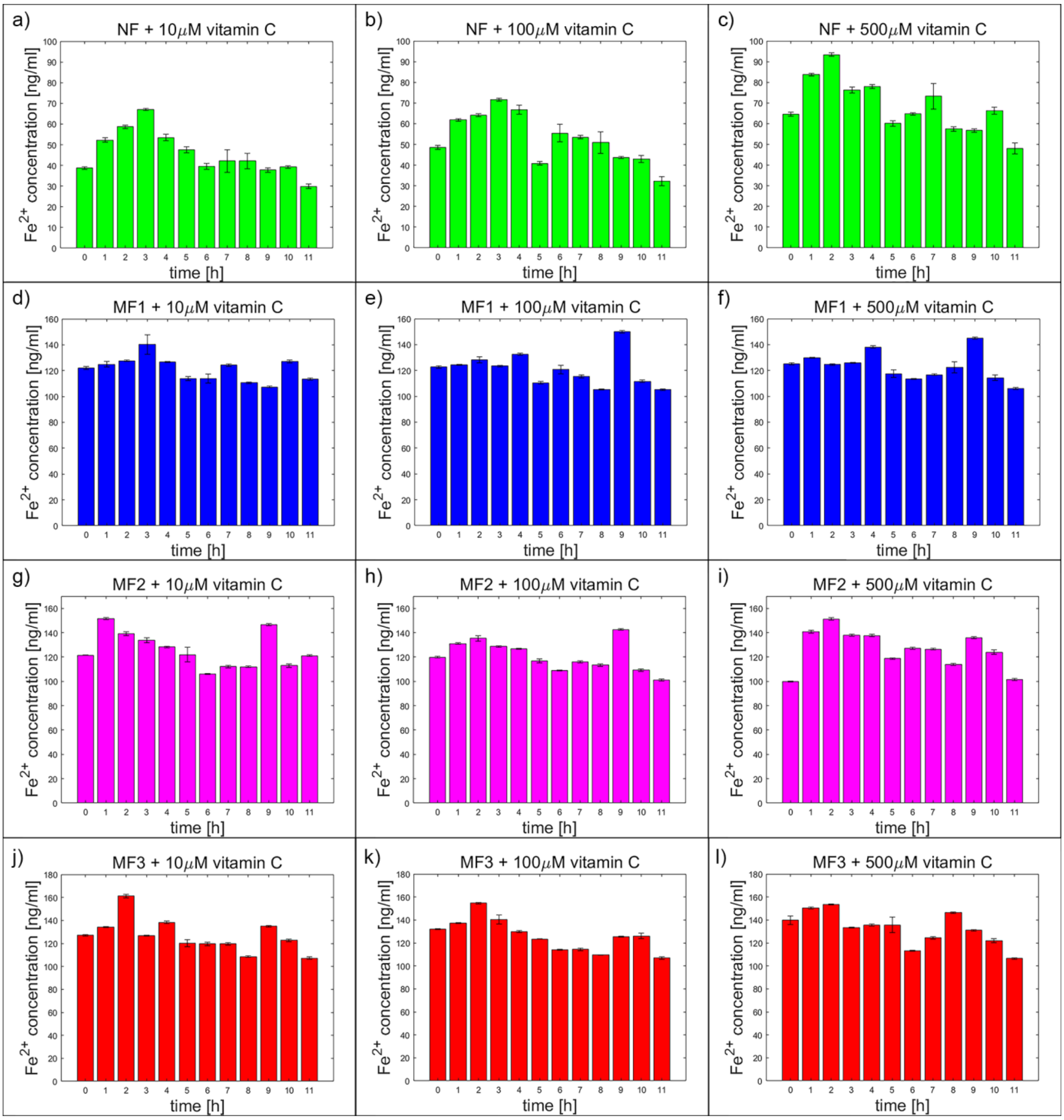

Figure S1. Time dependences of ferrous ion release (mean ± SD) from NF and MF with different LFs, induced by various concentrations of ascorbic acid (vitamin C):.(**a**)NF with LF = 868 and vitamin C concentration = 10 μM; (**b**)NF with LF = 868 and vitamin C concentration = 100 μM; (**c**) NF with LF = 868 and vitamin C concentration = 500 μM; (**d**)MF1 with LF = 553 and vitamin C concentration = 10 μM; (**e**)MF1 with LF = 553 and vitamin C concentration = 100 μM; (**f**) MF1 with LF = 553 and vitamin C concentration = 500 μM; (**g**)MF2 with LF = 733 and vitamin C concentration = 10 μM; (**h**)MF2 with LF = 733 and vitamin C concentration = 100 μM; (**i**) MF2 with LF = 733 and vitamin C concentration = 500 μM; (**j**) MF3 with LF = 873 and vitamin C concentration = 10 μM; (**k**)MF3 with LF = 873 and vitamin C concentration = 100 μM; (**l**); (**m**) MF3 with LF = 873 and vitamin C concentration = 500 μM.

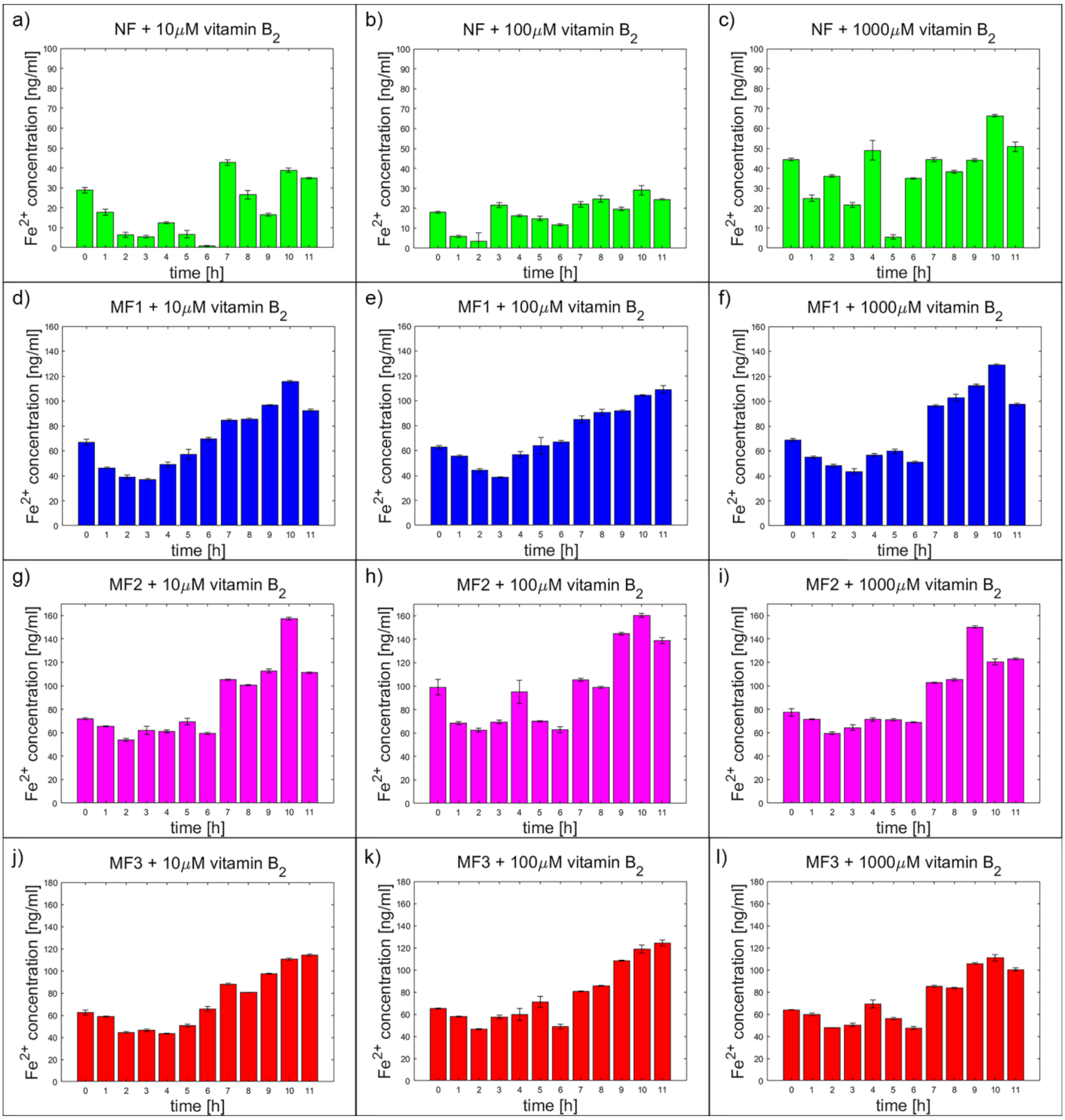

Figure S2. Time dependences of ferrous ion release (mean ± SD) from NF and MF with different LFs, induced by various concentrations of riboflavin (vitamin B_2): (**a**)NF with LF = 868 and vitamin B_2 concentration = 10 μM; (**b**) NF with LF = 868 and vitamin B_2 concentration = 100 μM; (**c**)NF with LF = 868 and vitamin B_2 concentration = 1000 μM; (**d**)MF1 with LF = 553 and vitamin B_2 concentration = 10 μM; (**e**) MF1 with LF = 553 and vitamin B_2 concentration = 100 μM; (**f**) MF1 with LF = 553 and vitamin B_2 concentration = 1000 μM; (**g**) MF2 with LF = 733 and vitamin B_2 concentration = 10 μM; (**h**)MF2 with LF = 733 and vitamin B_2 concentration = 100 μM; (**i**) MF2 with LF = 733 and vitamin B_2 concentration = 1000 μM; (**j**) MF3 with LF = 873 and vitamin B_2 concentration = 10 μM; (**k**)MF3 with LF = 873 and vitamin B_2 concentration = 100 μM; (**l**)MF3 with LF = 873 and vitamin B_2 concentration = 1000 μM.

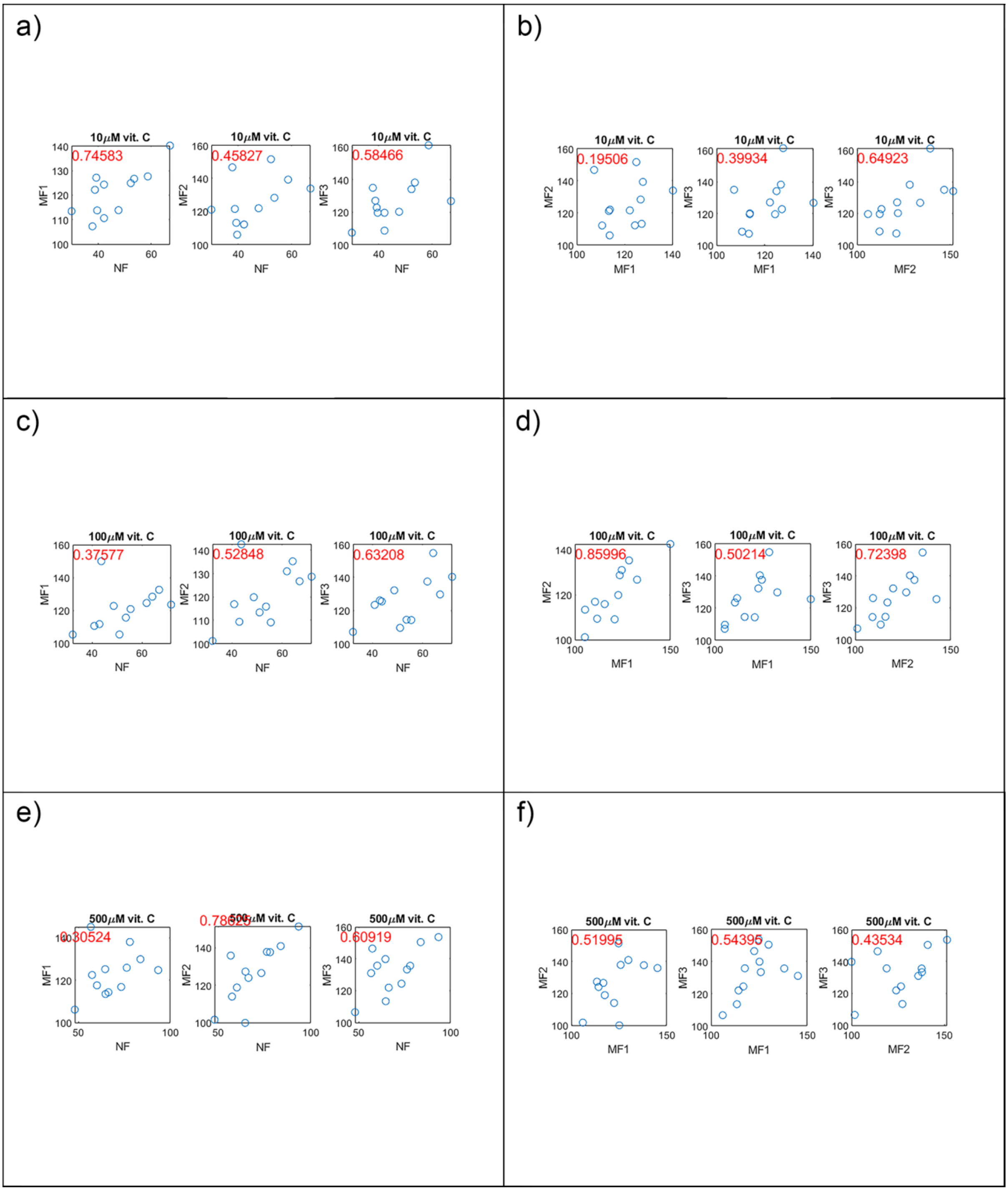

Figure S3. Correlation coefficient values (red) of ferrous ion release from NF and MF with different LFs, induced by various concentrations of ascorbic acid (vitamin C): (**a**) NF-MF1, NF-MF2, NF-MF3, vitamin C concentration = 10 μM; (**b**) MF1-MF2, MF1-MF3, MF2-MF3, vitamin C concentration = 10 μM; (**c**)NF-MF1, NF-MF2, NF-MF3, vitamin C concentration = 100 μM; (**d**) MF1-MF2, MF1-MF3, MF2-MF3, vitamin C concentration = 100 μM; (**e**)NF-MF1, NF-MF2, NF-MF3, vitamin C concentration = 500 μM; (**f**) MF1-MF2, MF1-MF3, MF2-MF3, vitamin C concentration = 500 μM.

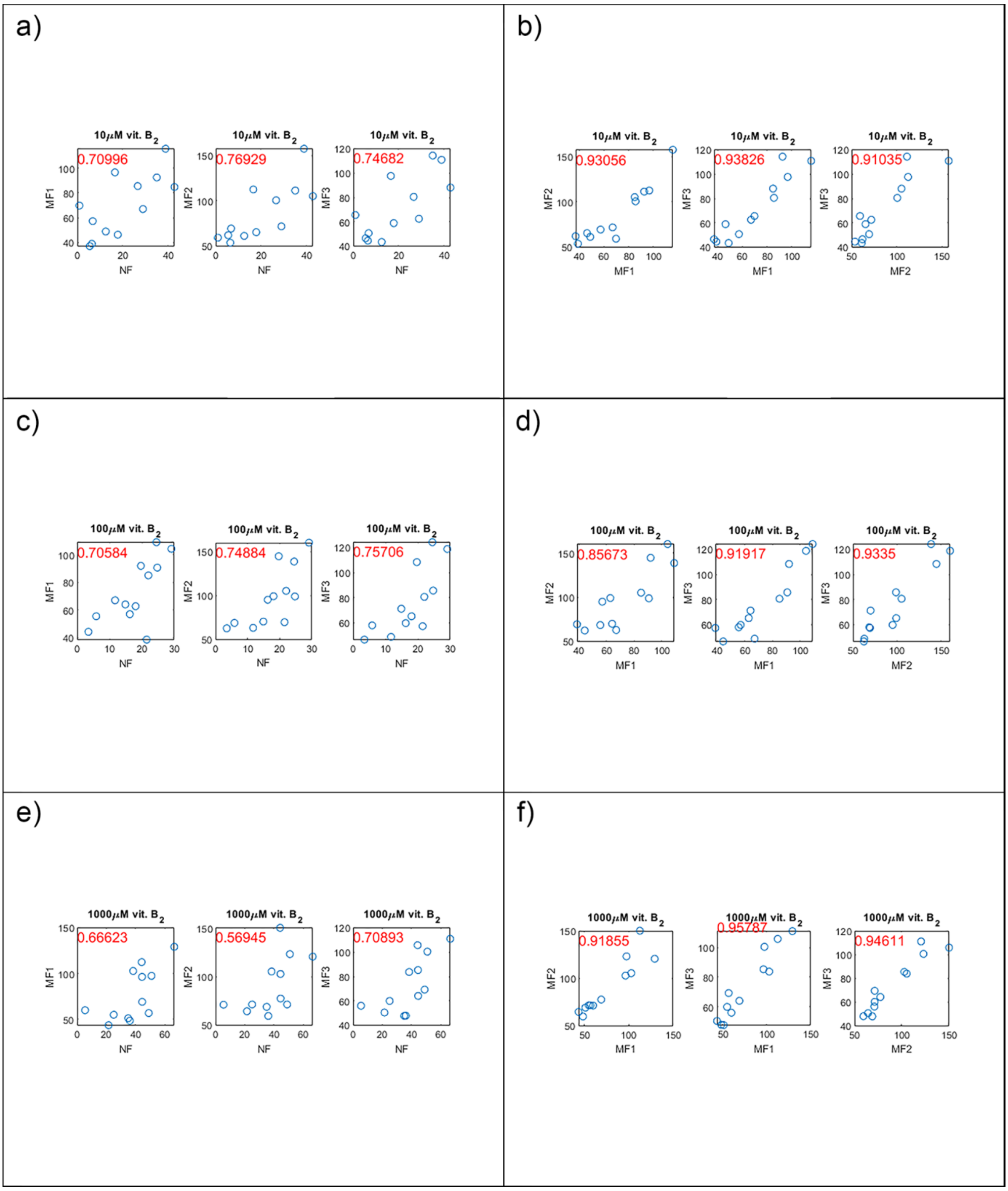

Figure S4. Correlation coefficient values (red) of ferrous ion release from NF and MF with different LFs, induced by various concentrations of riboflavin (vitamin B_2): (**a**) NF-MF1, NF-MF2, NF-MF3, vitamin B_2 concentration = 10 μM; (**b**) MF1-MF2, MF1-MF3, MF2-MF3, vitamin B_2 concentration = 10 μM; (**c**) NF-MF1, NF-MF2, NF-MF3, vitamin B_2 concentration = 100 μM; (**d**) MF1-MF2, MF1-MF3, MF2-MF3, vitamin B_2 concentration = 100 μM; (**e**)NF-MF1, NF-MF2, NF-MF3, vitamin B_2 concentration = 1000 μM; (**f**) MF1-MF2, MF1-MF3, MF2-MF3, vitamin B_2 concentration = 1000 μM.

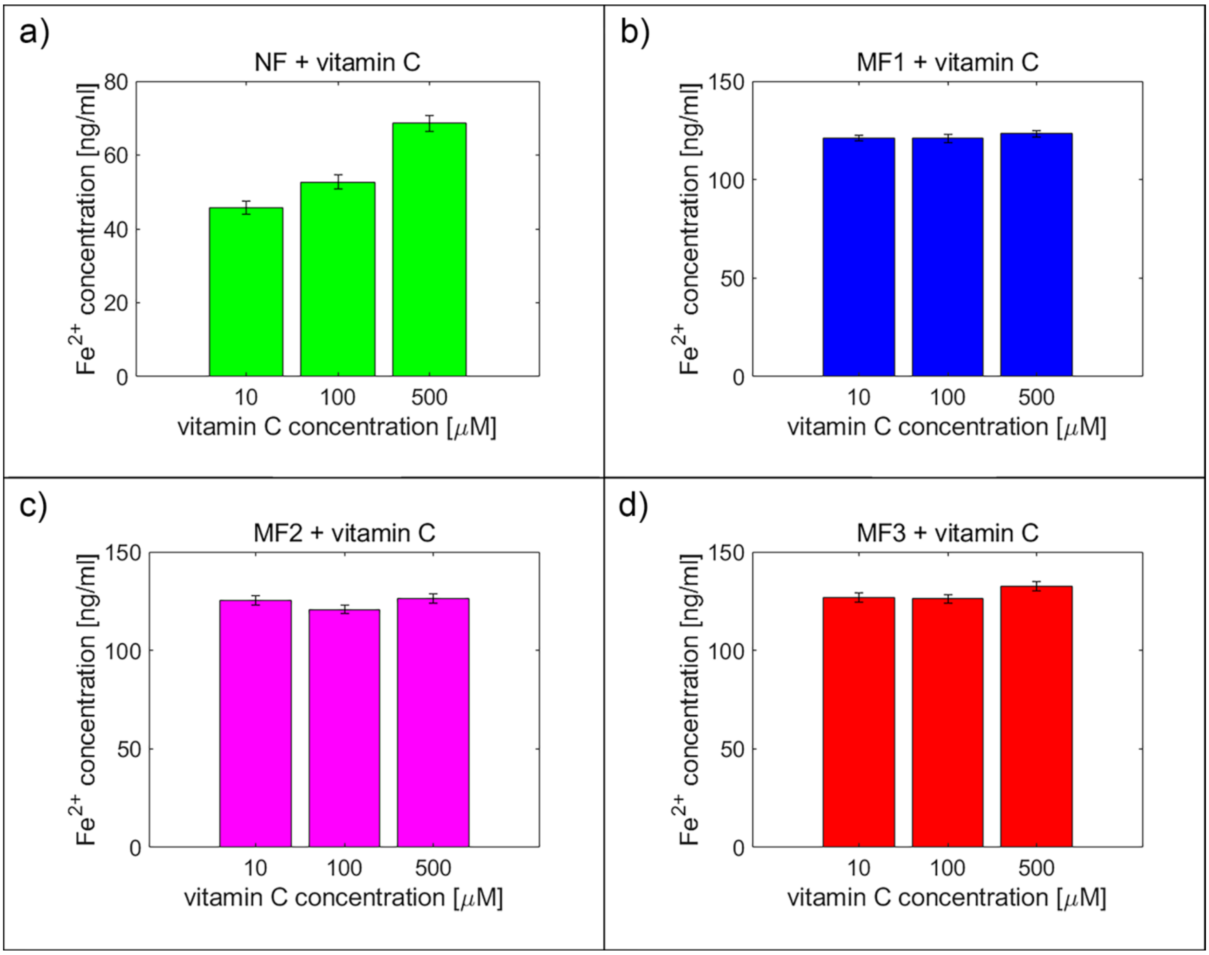

Figure S5. 95 % confidence interval (mean of the means ± 2 SEM) of the reduction rate of ferrous ions from NF and MF mineral cores per hour induced by various concentrations of riboflavin (vitamin C): (**a**) NF; (**b**) MF1; (**c**) MF2; (**d**) MF3.

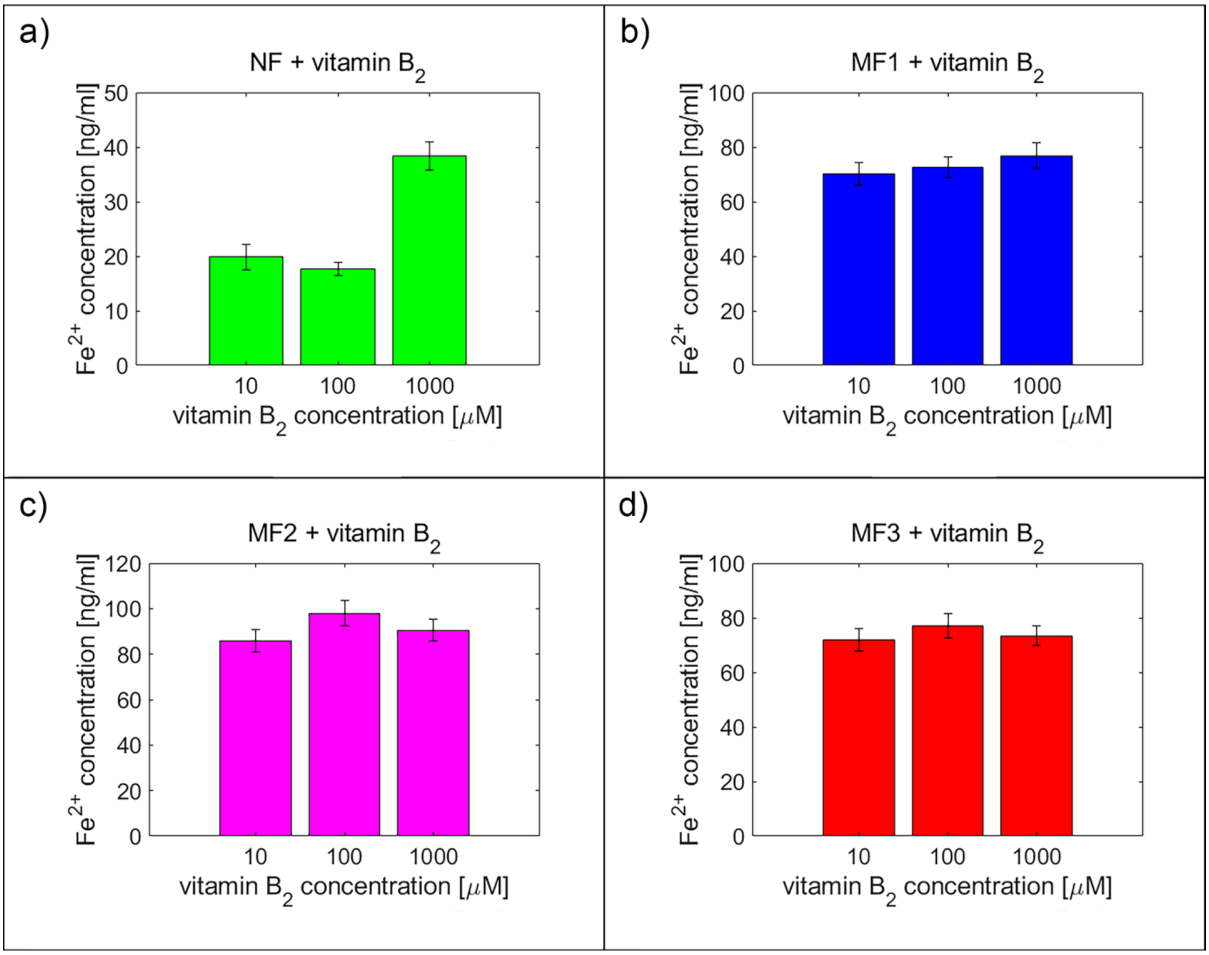

Figure S6. 95 % confidence interval (mean of the means ± 2 SEM) of the reduction rate of ferrous ions from NF and MF mineral cores per hour induced by various concentrations of riboflavin (vitamin B_2): (**a**) NF; (**b**) MF1; (**c**) MF2; (**d**) MF3.

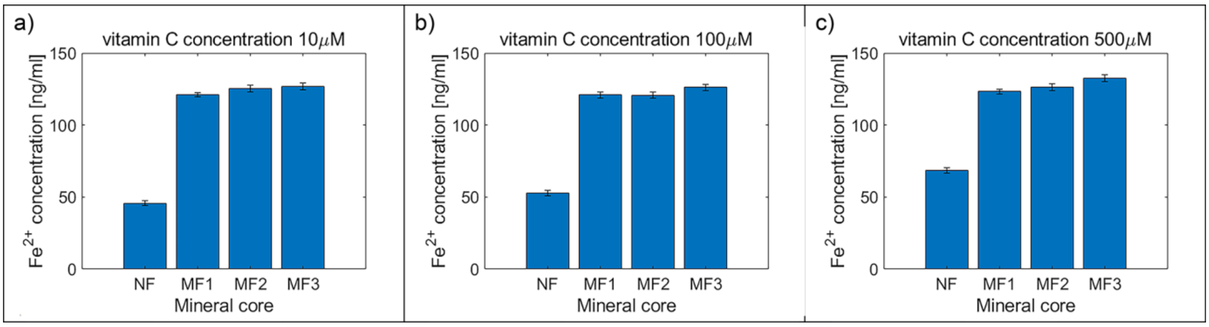

Figure 7S. 95 % confidence interval (mean of the means ± 2 SEM) of the reduction rate of ferrous ions from NF and MF mineral cores per hour induced by different concentrations of riboflavin (vitamin C): (**a**) 10 μM; (**b**) 100 μM; (**c**) 1000 μM.

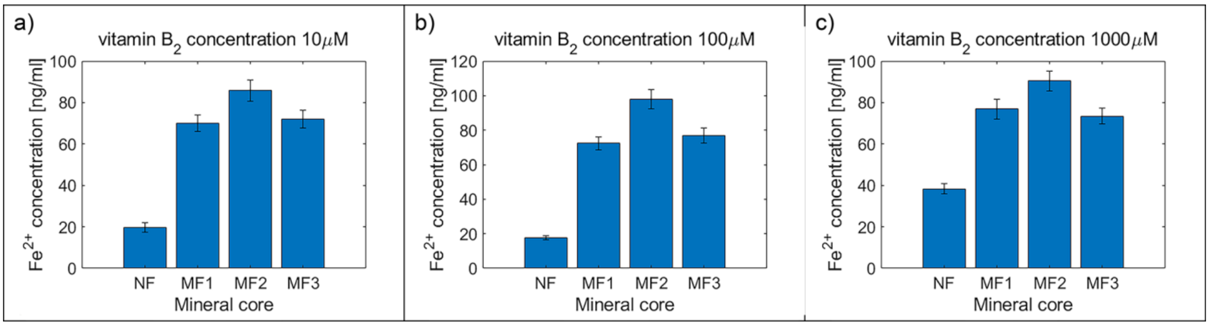

Figure S8. 95 % confidence interval (mean of the means ± 2 SEM) of the reduction rate of ferrous ions from NF and MF mineral cores per hour induced by different concentrations of riboflavin (vitamin B_2): (**a**) 10 μM; (**b**) 100 μM; (**c**) 1000 μM.
